# PI3K (Phosphatidylinositol 3-Kinase) Activation and Endothelial Cell Proliferation in Patients with Hemorrhagic Hereditary Telangiectasia Type 1

**DOI:** 10.3390/cells8090971

**Published:** 2019-08-24

**Authors:** Adriana Iriarte, Agnes Figueras, Pau Cerdà, José María Mora, Anna Jucglà, Rosa Penín, Francesc Viñals, Antoni Riera-Mestre

**Affiliations:** 1 HHT Unit, Hospital Universitari de Bellvitge, 08907 Barcelona, Spain; 2 Internal Medicine Department, Hospital Universitari de Bellvitge, 08907 Barcelona, Spain; 3 Bellvitge Biomedical Research Institute (IDIBELL), L’Hospitalet de Llobregat, 08907 Barcelona, Spain; 4 Program Against Cancer Therapeutic Resistance, Institut Catala d’Oncologia, Hospital Duran i Reynals, L’Hospitalet de Llobregat, 08907 Barcelona, Spain; 5 Oncobell Program, Bellvitge Biomedical Research Institute (IDIBELL), L’Hospitalet de Llobregat, 08907 Barcelona, Spain; 6 Dermatology Department, Hospital Universitari de Bellvitge, 08907 Barcelona, Spain; 7 Pathological Anatomy Department, Hospital Universitari de Bellvitge, 08907 Barcelona, Spain; 8 Physiological Sciences Department, Faculty of Medicine and Health Sciences, Universitat de Barcelona, L’Hospitalet de Llobregat, 08907 Barcelona, Spain; 9 Clinical Sciences Department, Faculty of Medicine and Health Sciences, Universitat de Barcelona, L’Hospitalet de Llobregat, 08907 Barcelona, Spain

**Keywords:** hemorrhagic hereditary telangiectasia, rare diseases, telangiectasia, transforming growth factor-beta (TGF-β), Smad pathway, phosphatidylinositol 3-kinase, mTOR inhibitors

## Abstract

Hemorrhagic hereditary telangiectasia (HHT) type 2 patients have increased activation of the phosphatidylinositol 3-kinase (PI3K) signaling pathway in telangiectasia. The main objective is to evaluate the activation of the PI3K pathway in cutaneous telangiectasia of HHT1 patients. A cutaneous biopsy of a digital hand telangiectasia was performed in seven HHT1 and eight HHT2 patients and compared with six controls. The study was approved by the Clinical Research Ethics Committee of our center. A histopathological pattern with more dilated and superficial vessels that pushed up the epidermis was identified in HHT patients regardless of the type of mutation and was associated with older age, as opposed to the common telangiectasia pattern. The mean proliferation index (Ki-67) was statistically higher in endothelial cells (EC) from HHT1 than in controls. The percentage of positive EC for pNDRG1, pAKT, and pS6 in HHT1 patients versus controls resulted in higher values, statistically significant for pNDRG1 and pS6. In conclusion, we detected an increase in EC proliferation linked to overactivation of the PI3K pathway in cutaneous telangiectasia biopsies from HHT1 patients. Our results suggest that PI3K inhibitors could be used as novel therapeutic agents for HHT.

## 1. Introduction

Hereditary hemorrhagic telangiectasia (HHT) or Rendu–Osler–Weber syndrome (ORPHA774) is a vascular autosomal-dominant germline disease, with an incidence of 1:6000 [1,2]. HHT is caused by mutations in genes involved in the transforming growth factor-beta (TGF-β) superfamily [3]. Mutations in endoglin (*ENG*) and activin A receptor type II-like 1 (*ACVRL1*) genes are detected in approximately 85% of cases submitted to molecular diagnosis for clinical suspicion of HHT, and cause HHT1 and HHT2, respectively [2,3]. Both proteins are specifically expressed in endothelial cells (ECs). Endoglin is an auxiliary co-receptor that promotes BMP9 signaling through the activin receptor-like kinase 1 (ALK1). Both proteins contribute to the signaling hub formed by BMP9–Endoglin–ALK1–Smad with high impact in EC proliferation, migration, and survival during angiogenesis [4]. The loss of function of endoglin and ALK1 proteins provokes an anomalous vascular overgrowth [4,5].

Although endoglin and ALK1 are components of the same BMP9 receptor complex, they are structurally and functionally different proteins and mutations in their genes are related with different clinical phenotypes [3,4]. Pulmonary and cerebral arteriovenous malformations (AVMs) are more common in patients with HHT1 and vascular hepatic malformations in those with HHT2 [6,7,8]. In spite of these differences in large vessels, telangiectasia is the characteristic lesion in the microvasculature in both HHT1 and HHT2 patients. Histopathologically, telangiectasia shows dilated post capillary venules directly connected with dilated arterioles losing the capillary bed [9]. These dilated microvessels are more prone to hemorrhage due to fragile walls and turbulent blood flow, especially those located in mucosae, such as nasal or gastrointestinal (GI) ones. Telangiectasia in nasal mucosae are the cause of spontaneous recurrent epistaxis, the earliest and most common clinical manifestation of HHT [6,7,8]. Limited data exist about the histological pattern of human cutaneous telangiectasia and possible differences between HHT1 and HHT2 patients [9].

Therapeutic strategies aim at reducing potential complications caused by vascular malformations, but there is currently no curative treatment for HHT [10,11]. Using heterozygous ALK1 mouse retinas and cultured ECs, we found that loss of ALK1 leads to increased EC proliferation as a result of the overactivation of PI3K (phosphatidylinositol 3-kinase) signaling. In the same study, mutations in ALK1 result in increased activation of PI3K signaling in human telangiectasia of patients with HHT2 compared with control vessels; furthermore, vascular retinal hyperplasia in a heterozygous ALK1 mouse model was rescued by pharmacological inhibition of PI3K activity [12]. Actually, the PI3K signaling pathway is involved in EC proliferation, migration, and survival by activating downstream of various angiogenic growth factors [13,14]. Taken together, these findings suggest a therapeutic intervention with PI3K pathway inhibitors, such as mTOR (mammalian target of rapamycin) inhibitors, for the treatment of HHT [12,14].

The goal of this work was to demonstrate the hyperactivation of the PI3K pathway in vascular lesions of patients with HHT1 compared with control vessels. Secondary objectives were to analyze endothelial proliferation in HHT1 patients compared to controls and to assess different histological patterns between HHT1 and HHT2 patients.

## 2. Materials and Methods

### 2.1. Study Design and Patients

Patients were selected from the referral HHT Unit at the Hospital Universitari de Bellvitge (Barcelona, Spain). This HHT unit serves adult patients from all over Catalonia (Spain), which has about seven million inhabitants. The inclusion criteria were having a definite diagnosis according to the Curaçao Criteria, mandatory cutaneous telangiectasia on the fingertip, and a positive genetic study for *ENG* mutations [15,16]. Seven HHT1 patients were included. Control samples were obtained from healthy skin in resection borders from melanomas. HHT2 patients were those used in our previous study on PI3K signaling pathway activation [12] and two new HHT2 patients. All selected patients gave their signed informed consent for telangiectasia biopsy in accordance with local ethics committee requirements. The study was approved by the Clinical Research Ethics Committee of the Hospital Universitari de Bellvitge (Barcelona, Spain; ethic approval number PR098/16).

### 2.2. Clinical Variables

Clinical characteristics at baseline and complementary tests were collected. Using the Curaçao criteria (recurrent epistaxis, cutaneous/mucosal telangiectasia, visceral involvement, and a first line family member with HHT), a diagnosis of HHT is considered “definite” if three or more criteria are present [16]. The severity of nosebleeds was measured according to the epistaxis severity score (ESS). Epistaxis is considered moderate or severe if ESS results are >4 or >7 points, respectively [17]. For the screening of pulmonary AVMs, a contrast transthoracic echocardiography (TTE) was performed [8,15]. The Barzilai scale was used to establish the degree of right–left shunt and the need for a thoracic computed tomography (CT) angiography to confirm the presence of pulmonary AVM [18]. In addition, an abdominal CT angiography was performed to study hepatic and/or abdominal AVMs. Hepatic involvement was defined according to the three classical patterns of abnormal vascular communications: Portovenous (from portal vein to hepatic vein), arteriovenous (from hepatic artery to hepatic vein), and arterioportal (from hepatic artery to portal vein) [8,19]. A GI endoscopic digestive study was performed according to guidelines, when there was disproportionate anemia to the degree of epistaxis or objectively confirmed overt GI bleeding [15]. Genetic tests were performed by the company Health in Code, S.L. (A Coruña, Spain) using next-generation sequencing [20].

### 2.3. Cutaneous Telangiectasia Biopsy

A punch biopsy (3 mm) from a cutaneous telangiectasia on the fingertip was obtained by a senior dermatologist under the usual conditions of sterility and hygiene. Samples were encrypted according to a code assigned to each patient. Biopsies were fixed in a formol buffer, dehydrated, and embedded in paraffin.

### 2.4. Histopathological Evaluation

Tissue sections (3 µm) were stained with hematoxylin and eosin for morphological analysis. To assess possible different histological patterns between HHT1 and HHT2 patients, we restained the samples from the six HHT2 human telangiectasia biopsies performed for our previous study on PI3K signaling pathway activation [12], plus two new HHT2 patients we were able to add to the series. Histological evaluation was performed by a senior pathologist.

### 2.5. Immunohistochemistry Studies

Tissue sections (3 µm) were stained by immunohistochemistry to determine the amount of expression of various proteins. Samples were deparaffinized in xylene and rehydrated in downgraded alcohols and distilled water. Antigen retrieval was performed under high-pressure conditions for 3 or 4 min in citrate buffer, pH 6 or 6.5, and incubated with 3% H_2_O_2_ for 10 min. Samples were then blocked with 1:20 goat serum for 1 h followed by incubation overnight at 4 °C with corresponding antibody.

In an attempt to describe the vessel area and to study the extracellular matrix, we performed immunohistochemistry with mouse monoclonal antibody anti-CD34 (Cat. #M7165; Dako, Carpenteria, CA, USA), an EC marker, and for mouse monoclonal antibody anti-Collagen IV (#M785; Dako), an extracellular component secreted by ECs. We also performed immunohistochemistry for monoclonal rabbit antibody anti–Ki-67 (SP6, Cat. MA5-15420; Thermo Fisher Scientific, Waltham, MA, USA), a proliferation marker, and for antiphospho-NDRG1 (Thr 346, Cat. #5482; Cell Signaling Technology, Inc., Beverly, MA, USA), polyclonal rabbit antiphospho-AKT antibody (Ser 473, Cat. #4060; Cell Signaling Technology, Inc.), and polyclonal rabbit antibody antiphospho-S6 (Ser 240/244, Cat. #2215; Cell Signaling Technology, Inc.), all markers of PI3K pathway activation. Sections were incubated with the specific secondary antibody, EnVision (Dako), followed by the DAB developing system (Dako). CD34 and pAKT were amplified with tyramide biotinXX reaction (Invitrogen #B40931, Thermo Fisher Scientific) and streptavidine–horseradish peroxidase (HRP) before DAB developing. Samples were counterstained with hematoxylin and visualized under light microscopy.

Three microscope images (200×) from each biopsy were used for vessels area analysis and collagen IV quantifications. Image J Software (developed at the National Institutes of Health and the Laboratory for Optical and Computational Instrumentation, University of Wisconsin, Madison, WI, USA) was used to measure the area of all CD34 immunostained vessels. Collagen IV was quantified on the higher amount zones for each vessel.

As a quality control and to confirm previous results of PI3K signaling pathway activation in HHT2 patients, we attempted to repeat all these immunohistochemistry studies in three HHT2 patients used in our previous study plus two new additional HHT2 samples [12]. For all samples, negative controls were performed, in which the section followed exactly the same protocol but in the absence of primary antibody.

### 2.6. Statistical Analysis

A descriptive statistical analysis was performed for all categorical and continuous variables expressed as proportions or means with standard deviations (SD), respectively. The statistical significance of group differences in continuous variables was determined using two-tailed Mann–Whitney U tests (*p* < 0.05) because no normal conditions were observed. The statistical significance of group differences in categorical variables was determined using two-tailed Fischer exact test (*p* < 0.05). Correlation was analyzed using two-tailed Pearson correlation (*p* < 0.01). Analyses were performed using SPSS, version 18 for the PC (SPSS, Inc., Chicago, IL, USA), and graphs were designed using GraphPad Prism (v5.0b, GraphPad Software, San Diego, CA, USA).

## 3. Results

### 3.1. Histopathological Vascular Pattern in Human Cutaneous Telangiectasia Biopsies

We analyzed cutaneous telangiectasia biopsies from seven patients with HHT1 with mutations in the *ENG* gene and from eight HHT2 patients with mutations in the *ACVRL1* gene. In HHT1 group, the mean age was 50.8 ± 10.2 years and four out of seven patients were female, while in HHT2 group, the mean age was 54.1 ± 9.2 years and four out of eight patients were female. All patients had a family history of HHT, and a definite diagnosis was made according to the Curaçao criteria. In our cohort, several types of variants were observed in the *ENG* gene: two nonsense, one frameshift (detected in three patients who were relatives), and one missense, as well as one large deletion involving exons 1–3 (copy number variant, CNV). In the *ACVRL1* gene, four pathogenic missense variants (one of them shared by three patients, including two relatives), one frameshift, and one in-frame deletion were identified.

In 71% of the HHT1 patients (five out of seven patients) pulmonary AVMs were identified and embolized, whereas in 87.5% of the HHT2 patients (seven out of eight patients) liver involvement was detected. Nosebleed severity measured by ESS was moderate (>4 points) in 57% of HHT1 patients and in 62.5% of HHT2 patients. The clinical characteristics of these patients are shown in Table 1.

**Figure 1:**
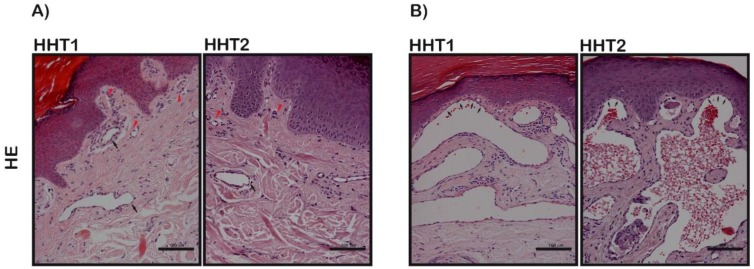
Hematoxylin and eosin (HE) microscope images. Scale bars, 100 µm. (**A**) Examples of conventional telangiectasia characterized by the presence of dilated microvessels at the superficial dermis (black arrows). Normal vessels are indicated with red arrows. One HHT1 patient and one HHT2. (**B**) Examples of more dilatated vessels expanding the papillary dermis between rete ridges that crowd up the epidermis layer (black arrows) (angiokeratoma-like pattern). One HHT1 patient and one HHT2.

These telangiectasia biopsies were assessed by a senior pathologist and there were no different histological patterns between HHT1 and HHT2. However, some differences were identified in the telangiectasia biopsies regardless of the type of mutation. One group resembled conventional telangiectasia characterized by the presence of dilated microvessels at the superficial dermis, while the others showed even more dilated vessels expanding the papillary dermis between rete ridges and pushing up the epidermis resembling an angiokeratoma-like pattern (Figure 1). We analyzed whether there were differences between these two histological subgroups and clinical data. We observed that patients with superficial dilated vessels were older (57.2 vs. 47.2 years; *p* = 0.042) and had higher nosebleed severity measured by ESS (5.4 vs. 4; *p* = 0.049) compared to patients with a conventional histological pattern (Table 2).

### 3.2. Vascular Size and Endothelial Cell Proliferation Are Increased in Cutaneous Telangiectasia Biopsies of Patients with HHT1 and HHT2 Compared to Controls

We analyzed cutaneous telangiectasia biopsies from seven patients with HHT1 and from six controls. The control group had a mean age of 60 ± 21.8 (range, 32–83) years and four (66.7%) were male. Unfortunately, in three of the six HHT2 patients used in our previous study, no sample was available to repeat immunohistochemistry studies.

**Figure 2:**
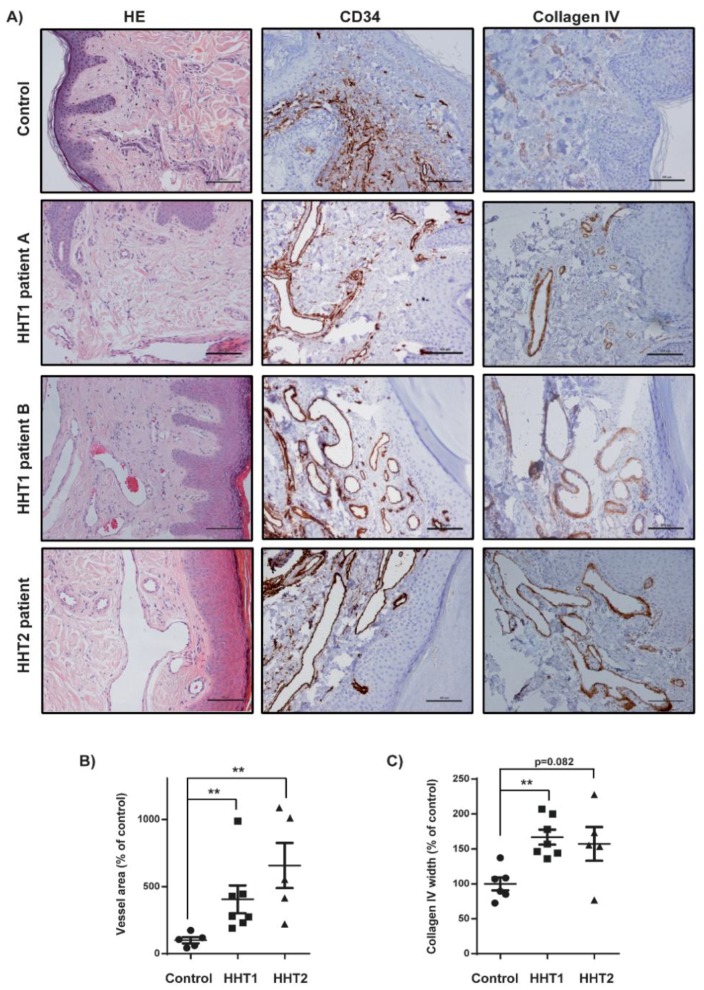
Increased vessels size and collagen IV staining in HHT1 and HHT2 cutaneous telangiectasia biopsies. (**A**) Hematoxylin and eosin (HE), CD34, and collagen IV staining of one control, two HHT1 patients and one HHT2 patient biopsies. Scale bars, 100 µm. (**B**) Quantification of the vessel area in controls (n = 6), HHT1 (n = 7), and HHT2 patients (n = 5). Results are represented as % relative to the control. Error bars indicate the standard error of the mean. Statistical significance of two-tailed Mann–Whitney U tests: ** *p* < 0.01. (**C**) Quantification of the collagen IV width in controls (n = 6), HHT1 (n = 7), and HHT2 patients (n = 5). Results are represented as % relative to the control. Error bars indicate the standard error of the mean. Statistical significance of two-tailed Mann–Whitney U tests: ** *p* < 0.01.

Patients with HHT1 and HHT2 were separately compared with controls. Both HHT1 and HHT2 showed significantly more enlarged vessels than controls when we measured the area of CD34-positive vessels. The basement membrane (BM) of these enlarged vessels was studied through collagen IV and we observed a higher presence of this marker in both HHT patients than in controls, though only statistically significant for HHT1 (Figure 2).

**Figure 3:**
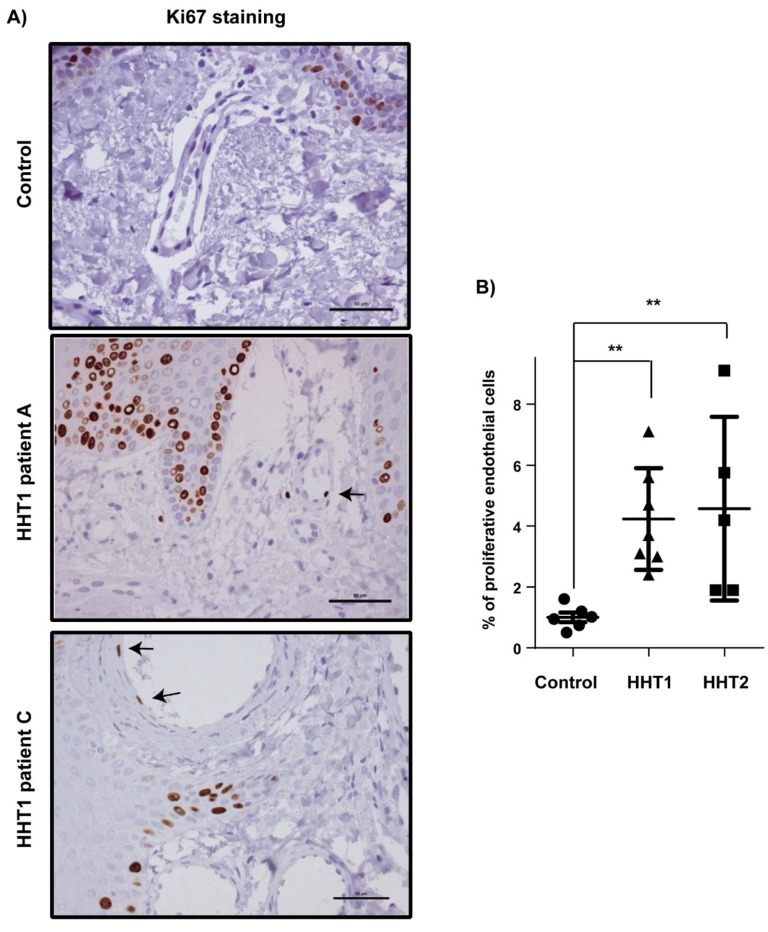
Increased endothelial cell proliferation in HHT1 cutaneous telangiectasia biopsies. (**A**) Ki-67 (brown nuclei, arrows) staining of endothelial cells in one control and two HHT1 patients. Scale bars, 100 µm. (**B**) Quantification of the percentage of Ki-67-positive endothelial cells in controls (n = 6), HHT1 patients (n = 7), and HHT2 patients (n = 5). Error bars indicate the standard errors of the mean. Statistical significance of two-tailed Mann–Whitney U tests: ** *p* < 0.01.

EC proliferation was analyzed by immunohistochemistry for Ki-67. The mean proliferation index was higher in ECs from HHT1 and HHT2 telangiectasia than in controls, statistically significant for both (Figure 3).

### 3.3. Overstimulation of the PI3K Pathway in Cutaneous Telangiectasia Biopsies of Patients with HHT1 Compared to Control Vessels

**Figure 4:**
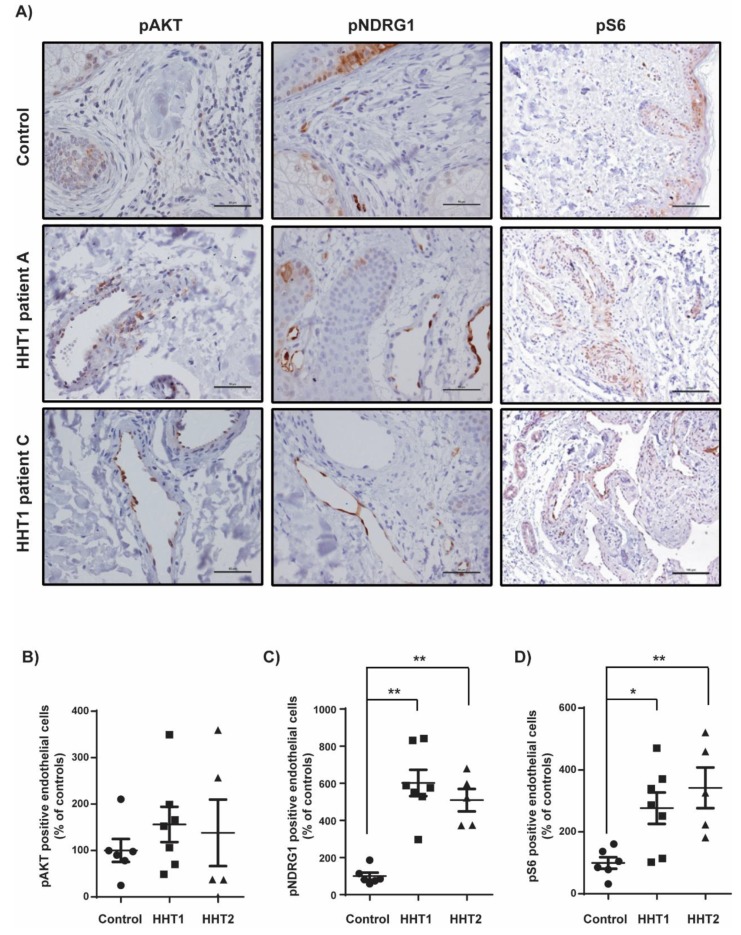
Increased activation of PI3K signaling in HHT1 cutaneous telangiectasia biopsies. (**A**) pAKT, pNDRG1, and pS6 staining of a control and two HHT1 patient biopsies. Scale bars, 100 µm. (**B**) Quantification of the percentage of pAKT-positive endothelial cells in controls (n = 6), HHT1 patients (n = 7), and HHT2 patients (n = 4). Results are represented as % relative to the control. Error bars indicate the standard error of the mean. (**C**) Quantification of the percentage of pNDRG1-positive endothelial cells in controls (n = 6), HHT1 patients (n = 7), and HHT2 patients (n = 5). Results are represented as % relative to the control. Error bars indicate the standard error of the mean. Statistical significance of two-tailed Mann–Whitney U tests: ** *p* < 0.01. (**D**) Quantification of the percentage of pS6-positive endothelial cells in controls (n = 6), HHT1 patients (n = 7), and HHT2 patients (n = 5). Results are represented as % relative to the control. Error bars indicate the standard error of the mean. Statistical significance of two-tailed Mann–Whitney U tests: * *p* < 0.05; ** *p* < 0.01.

To analyze PI3K pathway activation, we performed immunohistochemistry for phosphorylated proteins pNDRG1, pAKT, and pS6. The mean percentage of ECs positive for these markers resulted in higher values in HHT1 patients than in controls, being statistically significant for pNDRG1 (*p* = 0.001) and pS6 (*p* = 0.022). In accordance with previous results, HHT2 patients also showed statistically significant higher percentage of positive ECs than controls for pNDRG1 (*p* = 0.004) and pS6 (*p* = 0.004) (Figure 4).

We also compared the activation of PI3K pathway between HHT1 and HHT2 samples and no statistical differences were found. Moreover, we analyzed whether there were differences between those two histological subgroups mentioned above and PI3K pathway activation and we did not find significant differences.

## 4. Discussion

Notwithstanding that they are caused by mutations in different genes, both HHT1 and HHT2 share telangiectasia as the characteristic lesion in the microvasculature [5]. In fact, we have not found different histological patterns between HHT1 and HHT2 in human telangiectasia biopsies. The two patterns described were significantly correlated with age. Patients with more dilated vessels in contact with the epidermis (angiokeratoma-like pattern) were older and had a higher ESS than patients with typical smaller telangiectasia at the superficial dermis. Actually, age influences the natural history of HHT, as clinical manifestations in HHT are age-dependent [7,8,21]. Epistaxis usually worsened with age and GI bleeding usually began at the fifth or sixth decades of life [15,22]. These age-dependent bleeding manifestations could be explained by changes in telangiectasia, although other factors could also influence them. The fact that telangiectasia become larger and more superficial with age, even pushing up the epidermis as we observed, could make telangiectasia more prone to hemorrhage. Larger vascular malformations in the liver or the lungs also appear in adulthood, reflecting the active, lifelong angiogenesis process [8,15,21].

In our study, we have found an enlargement in vessels and a higher collagen IV expression in both HHT1 and HHT2 human telangiectasia biopsies than in controls. BMs are composed of macromolecules such as collagen IV [23]. In recent years, the understanding of the BM has changed from a mere structural component of tissues, to be considered an active modulator of blood vessel formation. Type IV collagen promotes cell adhesion, migration, differentiation, and growth, playing a critical role in EC proliferation and angiogenesis [23,24,25,26]. Moreover, in cutaneous collagenous vasculopathy, a benign rare acquired idiopathic microangiopathy characterized by diffuse telangiectasia, immunohistochemical staining revealed extensive deposition of type IV collagen around the blood vessels [27,28]. The exact role of type IV collagen in telangiectasia development in HHT, needs further investigation.

In the present study, we detected an increase in EC proliferation linked to an increase of the PI3K pathway activation compared to controls in cutaneous human telangiectasia biopsies from patients with HHT1. Similar results have been obtained in endoglin-null endothelial cells [29] and in endoglin-deficient animal models [5]. Endoglin and ALK1 are key components of the endothelial BMP9–Endoglin–ALK1–Smad hub signaling pathway that, in collaboration with the Notch signaling pathway, induces the maturation phase of the angiogenic process [4,5,30,31]. In this phase, ECs stop migration and proliferation, enter quiescence, produce extracellular matrix, and attract mural cells [12,30,31]. A decrease in the BMP9–Endoglin–ALK1–Smad axis, given by heterozygous mutations in *ENG* or *ACVRL1* genes, both converge in an increase in the PI3K pathway (and their readouts pAKT, pS6, and pNDGR1) that, in consequence, increases proliferation [4,5,13,30]. PI3K pathway inhibition improved vascular malformations in mouse models of HHT [14]. In addition, PI3K overstimulation has been shown to play a key role in other vascular malformations that also generate AVMs [32].

All these findings, together with similar results in our previous study with HHT2 patients, suggest that pharmacological block of the PI3K axis could be a therapeutic option for HHT disease [12,14]. mTOR inhibitors, such as sirolimus or everolimus, block the PI3K signaling pathway [33]. These drugs have shown efficacy in a phase II study for the treatment of patients affected by vascular malformations [34]. Concerning telangiectasia, sirolimus treatment also caused the regression of cutaneous and internal telangiectasias in a HHT patient, while treatment with the novel orally-available PI3K inhibitor BKM120 (also named buparlisib) caused a decrease in epistaxis in a HHT2 patient with ovarian cancer [35,36]. mTOR inhibitors have been thoroughly tested as immunosuppressant agents and are specially recommended in patients with liver transplantation for hepatocellular carcinoma [37]. However, optimal doses and the long-term benefit/risk ratio of these drugs for the treatment of HHT patients are unknown. Because loss-of-function mutations in *ALK1* also cause vascular overgrowth, some authors aimed at activating this pathway as a therapeutic value. In a recent study, tacrolimus (FK-506) activated Smad1/5/8 and opposed to the pro-angiogenic gene expression associated with *ALK1* loss-of-function in human umbilical vein ECs (HUVECs) and prevented the hypervascularization in a BMP9/10-immunodepleted retina mouse model [38]. Further investigation is needed to determine whether concomitant use of both mTOR–PI3K inhibitors and ALK1 activators provides additional benefit in the treatment of HHT patients.

## 5. Conclusions

In conclusion, besides the conventional histological telangiectasia appearance we have identified an angiokeratoma-like pattern that could be related to age. Human HHT1 and HHT2 telangiectasia biopsies showed higher collagen IV deposition than controls. Immunohistochemical staining revealed an increase in endothelial cell proliferation linked to an increased activation of the PI3K pathway in HHT1. Our results suggest that PI3K inhibitors (or mTOR inhibitors) could be used as novel therapeutic agents for both HHT1 and HHT2 patients.

## Figures and Tables

**Table 1:** Hereditary hemorrhagic telangiectasia type 1 and 2 patient characteristics.

No.	Age, Years	M/F	TTE	Thoracic CT	Abdominal CT	GI Telangiectasia	CI, L/min/m^2^	ESS	Mutations
1	66	F	2	Pulmonary AVM RUL, LUL, LLL (embolized)	Pancreatic telangiectasias	Esophagus–duodenum	3.06	4.75	*ENG*: Exon 6: p.(Tyr258*) (c.774C > A) NONSENSE
2 *	52	F	1	Pulmonary AVM LLL (embolized)	No pathological findings	Gastroduodenal, proximal–middle jejunum	2.47	3.04	*ENG*: Exon 7: p.(Val323Leufs*10) (c.967_968delGT) FRAMESHIFT
3	59	F	2	Pulmonary AVM LUL, RLL, RUL (embolized)	Ileal-jejunum AVM	Stomach–proximal–middle jejunum	2.32	7.68	*ENG*: Exon 3: p.(Arg93*) (c.277C > T) NONSENSE
4	38	M	3	Pulmonary AVM LLL, RUL (embolized)	Intrahepatic telangiectasias Hepatic AP shunt	Not performed	3.4	3.84	*ENG*: Exon 9: p.(Cys412Tyr) (c.1235G > A) MISSENSE
5 *	43	M	3	Pulmonary AVM LUL, RUL (embolized)	No pathological findings	Not performed	2.44	4.42	*ENG*: Exon 7: p.(Val323Leufs*10) (c.967_968delGT) FRAMESHIFT
6	42	M	1	No pathological findings	Intrahepatic telangiectasias	Stomach–ascending colon	2.77	3.33	*ENG*: Exon 1–3: (c.-3659_361-537del) CNV (COPY NUMBER VARIANT)
7 *	56	F	2	No pathological findings	No pathological findings	Gastroduodenal–Ileocecal valve	3.36	5.47	*ENG*: Exon 7: p (Val323Leufs*10) (c.967_968delGT) FRAMESHIFT
8 ^+^	62	F	1	No pathological findings	Intrahepatic telangiectasias Hepatic AV shunt Hepatic artery enlargement FNH Intrapancreatic AVM Ileal AVM Cecal AVM Uterine AVM	Not performed	3.74	2.83	*ACVRL1*: Exon 3: p.(Thr82del) (c.244_246delACC) IN-FRAME DELETION
9	49	M	1	No pathological findings	No pathological findings	Not performed	2.28	6.38	*ACVRL1*: Exon 3: p.(Cys77Arg) (c.229T > C) MISSENSE
10 **	41	F	3	Pulmonary AVM LLL	Intrahepatic telangiectasias Hepatic AV shunt Hepatic artery enlargement NRH Uterine AVM	Not performed	3.12	2.93	*ACVRL1*: Exon 10: p.(Arg479Pro) (c.1436G > C) MISSENSE
11 **^,+^	70	F	0	No pathological findings	Hepatomegaly Hepatic AV shunt Hepatic artery enlargement Intrapancreatic AVM Left renal artery aneurysm	Not performed	3.7	6.59	*ACVRL1*: Exon 10: p.(Arg479Pro) (c.1436G > C) MISSENSE
12	49	M	0	Not performed	Intrahepatic telangiectasias Intrapancreatic AVM Gastro-omental artery aneurysms	Not performed	4.57	13.5	*ACVRL1*: Exon 10: p.(Arg479Pro) (c.1436G > C) MISSENSE
13 ^+^	51	M	1	No pathological findings	Hepatomegaly Hepatic AP shunt Hepatic artery enlargement	Not performed	3.3	6.05	*ACVRL1*: Exon 10: p.(Arg484Trp) (c.1450C > T) MISSENSE
14	51	F	1	No pathological findings	Hepatic telangiectasias Hepatic AP shunt Hepatic AV shunt	Not performed	3.2	1.41	*ACVRL1*: Exon 3: p.(Pro23Leufs*2) (c.68delC) FRAMESHIFT
15	60	M	0	No pathological findings	Hepatic telangiectasias Hepatic AP shunt Hepatic AV shunt Intrapancreatic AVM	Gastroduodenal	2.9	6.09	*ACVRL1*: Exon 3: p.(Arg67Gln) (c.200G > A) MISSENSE

Contrast transthoracic echocardiography (TTE). Computed tomography (CT). Arteriovenous malformation (AVM). Cardiac index (CI). Epistaxis severity score (ESS). Female (F). Right upper lobe (RUL), left upper lobe (LUL), left lower lobe (LLL), right lower lobe (RLL). AP indicates arterioportal (hepatic artery to portal vein); AV, arteriovenous (hepatic artery to hepatic vein); Focal nodular hyperplasia (FNH); Nodular regenerative hyperplasia (NRH); * Patients 2, 5, and 7 are relatives. ** Patient 11 is the mother of patient 10. ^+^ HHT2 patients already enrolled in our previous study with available sample to repeat PI3K immunohistochemistry studies [12].

**Table 2:** Clinical characteristics of HHT patients according to different histological patterns.

	Conventional Pattern n (%) or Mean (SD)	Angiokeratoma-Like Pattern n (%) or Mean (SD)	P
Patients	7 (46.6)	8 (53.3)	NA
Female gender	3 (42.8)	5 (62.5)	0.619
Age, years	47.2 (9.5)	57.2 (7.0)	0.042 *
Mutation			
*ENG*	5 (87.5)	2 (25)	0.132
*ACVRL1*	2 (28.5)	6 (75)	
ESS	3.9 (0.6)	5.4 (1.5)	0.049 *

Standard deviation (SD); endoglin (*ENG*); activin (*ACVRL1*); epistaxis severity score (ESS); * *p* < 0.05.
